# Gelatin-Graphene Oxide Nanocomposite Hydrogels for *Kluyveromyces lactis* Encapsulation: Potential Applications in Probiotics and Bioreactor Packings

**DOI:** 10.3390/biom11070922

**Published:** 2021-06-22

**Authors:** Jorge Luis Patarroyo, Eduardo Fonseca, Javier Cifuentes, Felipe Salcedo, Juan C. Cruz, Luis H. Reyes

**Affiliations:** 1 Grupo de Diseño de Productos y Procesos (GDPP), Department of Chemical and Food Engineering, Universidad de los Andes, Bogotá 111711, Colombia; jl.patarroyoa@uniandes.edu.co (J.L.P.); ea.fonseca10@uniandes.edu.co (E.F.); fesalced@uniandes.edu.co (F.S.); 2 Department of Biomedical Engineering, Universidad de Los Andes, Bogotá 111711, Colombia; jf.cifuentes10@uniandes.edu.co

**Keywords:** graphene oxide, double-crosslink, encapsulation, probiotics, packings

## Abstract

Nutraceutical formulations based on probiotic microorganisms have gained significant attention over the past decade due to their beneficial properties on human health. Yeasts offer some advantages over other probiotic organisms, such as immunomodulatory properties, anticancer effects and effective suppression of pathogens. However, one of the main challenges for their oral administration is ensuring that cell viability remains high enough for a sustained therapeutic effect while avoiding possible substrate inhibition issues as they transit through the gastrointestinal (GI) tract. Here, we propose addressing these issues using a probiotic yeast encapsulation strategy, *Kluyveromyces lactis*, based on gelatin hydrogels doubly cross-linked with graphene oxide (GO) and glutaraldehyde to form highly resistant nanocomposite encapsulates. GO was selected here as a reinforcement agent due to its unique properties, including superior solubility and dispersibility in water and other solvents, high biocompatibility, antimicrobial activity, and response to electrical fields in its reduced form. Finally, GO has been reported to enhance the mechanical properties of several materials, including natural and synthetic polymers and ceramics. The synthesized GO-gelatin nanocomposite hydrogels were characterized in morphological, swelling, mechanical, thermal, and rheological properties and their ability to maintain probiotic cell viability. The obtained nanocomposites exhibited larger pore sizes for successful cell entrapment and proliferation, tunable degradation rates, pH-dependent swelling ratio, and higher mechanical stability and integrity in simulated GI media and during bioreactor operation. These results encourage us to consider the application of the obtained nanocomposites to not only formulate high-performance nutraceuticals but to extend it to tissue engineering, bioadhesives, smart coatings, controlled release systems, and bioproduction of highly added value metabolites.

## 1. Introduction

The efficient oral administration and subsequent absorption of different bioactive compounds such as drugs, proteins, peptides, hormones, and antibodies by the human body have been widely studied due to various challenges to preserve their functionality and structural integrity as they transit through harsh environments in the gastrointestinal (GI) tract [1,2,3]. Similarly, nutraceuticals, which generally are poorly soluble in water, have low bioavailability by this route of administration [4]. This is also the case of whole cells, including lactic acid bacteria (LAB), *Bifidobacteria*, *Escherichia coli* Nissle 1917, and beneficial molecules such as bivalent fusion protein r-BL with recombinant protein U-Omp19, *Garcinia mangostana* L. ethanolic extract, and insulin [5,6,7,8,9,10,11]. As these bioactive molecules reach the small intestine lumen, they need to come across an extracellular mucus layer to finally reach the surface of the brush border for absorption [12]. Such a physiological barrier poses a significant obstacle for absorption of administered compounds since the thickness varies from 30 to 300 µm, increasing from the intestine to the rectum [12]. Consequently, only a tiny fraction (tight junction proteins allow the exchange of molecules with molecular weight < 500 Da [13]) made it to the systemic circulation. For these reasons, it is of paramount importance to design encapsulating materials that not only can protect the cargoes but transiently maintain high levels of the bioactive compounds as they transit the lower portion of the GI tract.

Regarding the upper portion of the GI tract, the oral cavity is the first compartment where encapsulates are subjected to mechanical stresses and enzymatic degradation conditions provided by saliva. The transit is slow, and the cell viability impact is imperceptible [12]. The stomach is the second compartment where encapsulates are exposed to harsh conditions. This organ is responsible for breaking down food into smaller and more assimilable molecules [14]. This occurs under an acidic environment, which, when in contact with bioactive compounds, will induce protonation of some pendant groups such as phospholipids, amino and carboxyl groups [6], and the decrease of cell cytoplasmic pH. Subsequently, the molecules reach the small intestine, where they encounter a thick mucus layer that protects the epithelial tissue cells against various mechanical effects, mainly attributed to the intestinal transit. At the same time, this layer maintains the cells relatively isolated from the ingested nutritious or pharmacological substances. Therefore, they need to overcome this extra resistance to transport prior to absorption [12]. Mucus provides a physical barrier since it is formed by a negatively charged fibrous mesh. For this reason, electrostatic interactions with bioactive compounds might still be possible and even help them to remain attached to the intestinal lumen as they start diffusing across the mucus layer [14].

Encapsulation of bioactive compounds or whole cells for oral administration has been achieved with the aid of several materials, including collagen, gelatin, alginate, chitosan, gum Arabic, maltodextrin, starch, sodium caseinate, polyvinyl alcohol, polyethylene glycol, and polyacrylic acid [15,16,17,18,19]. Polymeric materials, particularly hydrogels, have been described as the preferred choice due to characteristics such as hydrophilic porous matrix, flexibility, high biocompatibility and biodegradability, prolonged consistency, user-friendliness, low cost, and ease of access [20,21,22]. To aid in finding its optimal parameters, there have been several experimental and in silico studies that confirm a strong dependency on solubility, high degree of functional design space, surface multivalency, facile chemical modification, high stability, and ease of integration with other materials such as lipids and nanoparticles [23,24,25,26]. For instance, Mdlovu et al. designed a magnetic nanocarrier composed of iron oxide magnetic nanoparticles coated with Pluronic F127 for the delivery of the chemotherapeutic drug doxorubicin (DOX) in neuroblastoma where the drug release profile showed a pH-dependent drug release where more DOX was released under acidic environments than in neutral conditions [27]. Most recently conducted studies have attempted developing smart hydrogels capable of responding to environmental stimuli to release the therapeutic cargoes. Such specific responsiveness (e.g., changes in volume, size, structural conformation, and ionic charge) takes place by changes in pH, temperature, presence of enzymes, light irradiation, application of electric and magnetic fields [28,29,30,31,32]. For instance, Zhang et al. developed a polyvinyl alcohol (PVA)/poly(2-(N,N′-dimethylamino) ethyl methacrylate) (PDMAEMA)-poly (acrylic acid) (PAAc) hydrogel that showed a pH-responsive swelling behavior and controlled equilibrium swelling ratio due to both the DMAEMA and AAc in the network [33]. Finally, hydrogels have been designed to incorporate the attributes needed to mimic the biological behavior of the target tissues in a living organism [34,35]. For instance, Puertas-Bartolomé et al. reported preparing a hybrid system of hydrogels and catechol to serve as a bioactive wound dressing. Results revealed that the material exhibits high biocompatibility and stability, normal inflammatory responses, and faster vascularization, making it a suitable extracellular matrix-mimic platform with high cell affinity and bioactive functionalities [34].

The widespread and sometimes misguided use of antibiotics in a large portion of the population has led to the increasing emergence of pathogens with a worrisome resistance to such molecules [36,37]. In contrast, other microorganisms play a fundamental role in developing and strengthening the immune system against such pathogens. To take advantage of this potential, numerous live biotherapeutic products (LBPs), termed probiotics, have been developed recently, which not only offer the possibility to counteract pathogens but also to bring other health benefits, including restoring the gut microflora, providing immune modulation, maintaining bone health, relieving lactose intolerance, and decreasing the levels of LDL cholesterol [38,39,40]. For instance, *Lactobacillus* can generate innate and adaptive immune responses, produces antimicrobial substances, interacts with both intestinal epithelial cells (IECs) and dendritic cells (DCs), prevents pathogen colonization and proliferation, and activates antigen-specific response [41]. These live therapeutic agents must remain viable from the moment they are administered orally until they reach their target of interest in the small intestine [38].

Another emerging platform for the delivery of bioactive compounds are nanomaterials (polymeric nanoparticles, liposomes, bio-nanoparticles, micelles, peptides, metallic nanoparticles, and carbon-based nanomaterials [42,43]). Much progress has been made to achieve specific delivery goals regarding selectivity, bioavailability, and guided transport and targeting [44,45,46]. For instance, carbon-based nanomaterials such as graphene oxide, reduced graphene oxide, graphene quantum dots, graphene nanoribbons, silica-based nanocarriers, and inorganic nanoparticles [42,43,47,48] have been used to infiltrate tumors with the aid of cell-penetrating agents, and due to enhanced permeation and retention (EPR) mechanisms, they remain inside them for a more extended period [49,50,51].

Recent studies have started to combine the most prominent attributes of hydrogels and nanomaterials by synthesizing nanocomposites. Through this approach, the formulated delivery carriers for both bioactive compounds and whole microorganisms showed enhanced fundamental physicochemical properties, including small size, ability to form a stable complex, high drug loading, reduced toxicity, and improved mechanical strength [52,53,54]. For instance, in a recent study by Ghibaudo et al., pectin was combined with iron oxide nanoparticles and succeeded in fulfilling two essential functions: protecting probiotic lactic acid bacteria from simulated gastrointestinal tract fluids and delivering iron safely to the intestine [55].

An emerging nanomaterial that revolutionizes different industries due to its unique properties (e.g., high thermal and electrical conductivity, stability, chemical inertness, impermeability [56,57,58]) is graphene. Making more stable and smarter hydrogels is not the exception. Several reports have discussed the use of graphene to alter properties such as transparency and absorbance, particle stability, biocompatibility, toxicity, and the ability to mimic extracellular matrix and tissue microenvironments [59]. Despite its enormous potential, graphene exhibits a few drawbacks, including limited solubility in aqueous media and the absence of a well-studied toxicology profile [60]. In this regard, a much better alternative to graphene for preparing mixtures in aqueous media (which is the case for most biological and biomedical applications) is graphene oxide (GO) [59]. This nanomaterial is obtained from graphene by chemical oxidation of natural graphite [61]. Additionally, it has been reported to exhibit high loading capacity of drug-like molecules, large surface area, negative electric charge, and the presence of surface functional groups such as hydroxyl, epoxy, carbonyl, and carboxylic [62], which might enhance colloidal stability [63] and enable different bioconjugation routes to other nanomaterials, polymers and drugs [64]. Modification of hydrogels by GO aims to improve the material’s mechanical response (tensile and compression strength resistance) and generate a system for controlled loading, delivery of drugs or bioactive agents [65], and tissue engineering [66]. In all cases, GO has been included in formulations where the main bioactive compounds need significant stability and protection against external environmental factors that might induce their degradation and eventually their loss of functionality [67]. Based on the attractive attributes of GO, we decided to extend our previous work on the encapsulation of *Kluyveromyces lactis* [68] in gelatin hydrogels to GO-modified materials. We hypothesized that the modified encapsulates significantly improve their simulated GI tract media performance concerning the unmodified ones. Although GO has been investigated previously in biomedical applications, there is no consensus about its biocompatibility. However, most studies appear to coincide that it depends strongly on the final GO concentration in the hydrogel or biomaterial. In this regard, composites with GO concentrations in the range of low to moderate (i.e., between 0.1 and 0.25 mg in mice) tend to exhibit an imperceptible impact on biocompatibility. Therefore, this study is dedicated to investigating variations in the thermal, biological, and mechanical responses and the microstructural organization of gelatin hydrogels after modification with different GO concentrations. Our goal is to improve the encapsulates’ performance in preserving the probiotics’ activity as they encounter separate compartments along the GI tract.

## 2. Materials and Methods

### 2.1. Microorganism and Culture Media

**Figure 1:**
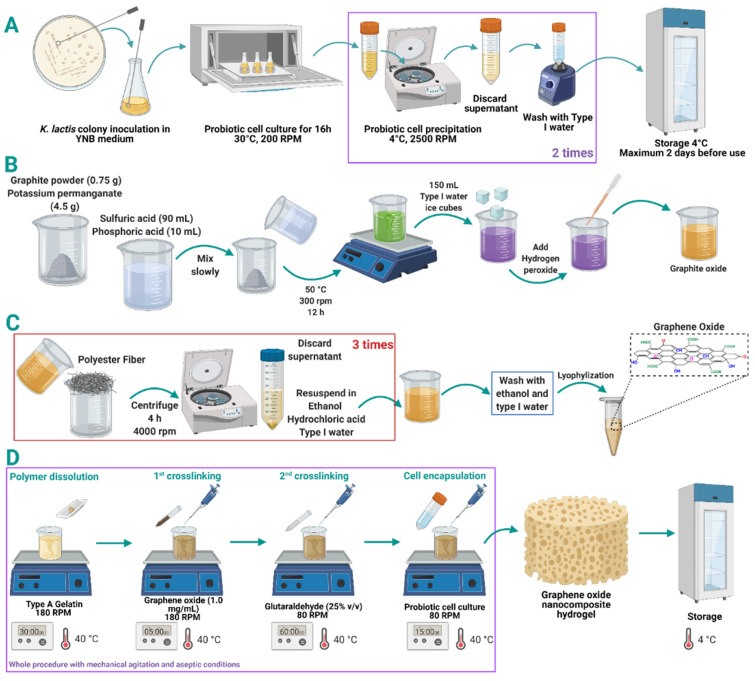
*K. lactis* cell inoculation, culture, washing, and storage for graphene oxide (GO) nanocomposite hydrogels encapsulation (**A**). Synthesis of GO through the modified Tour’s method. (**B**) Oxidation reaction and (**C**) washing process. Protocol for encapsulation of *K. lactis* probiotic cells in the matrix of gelatin-GO nanocomposite hydrogels (**D**).

As in our previous work [68], *Kluyveromyces lactis* GG799 from *K. lactis* Protein Expression Kit (New England Biolabs, Ipswich, MA, USA) was the probiotic strain studied. It was proliferated in YPGlu plates (yeast extract 1.0% (*w*/*v*), peptone 2.0% (*w*/*v*), glucose 2.0% (*w*/*v*), agar 1.5% (*w*/*v*), ampicillin 100 µg/mL) and grown in liquid medium (yeast nitrogen base (YNB) 0.68% (*w*/*v*), glucose 2.0% (*w*/*v*), lactose 2.0% (*w*/*v*), L-histidine 0.001% (*w*/*v*)) at 30 °C and 200 RPM overnight. After that, the culture was centrifuged at 2500 RPM for 10 min at 4 °C to obtain and register the biomass weight. Finally, probiotic yeast cells were washed twice with water and stored at 4 °C. Details are presented in Figure 1. The fermentation medium was the same liquid medium supplemented with lactose (16% (*w*/*v*)) to a final bioreactor operation volume of 170 mL.

### 2.2. Synthesis and Characterization of Graphene Oxide (GO)

Graphene oxide (GO) was synthesized following a modified version of the Tour’s method previously described by Marcano and colleagues [69]. In brief, 90 mL of sulfuric acid and 10 mL of phosphoric acid were mixed and slowly added to 0.75 g of graphite powder and 4.5 g of potassium permanganate. The resulting solution was left to react at 50 °C under constant stirring for 12 h. Next, 150 mL of type I water ice cubes were added to stop the oxidation. Then, hydrogen peroxide was added dropwise until a visual change was observed (from dark purple to yellow). Next, the solution was filtered with polyester fiber and centrifuged at 4000 rpm for 4 h, supernatants were discarded, and pellets were resuspended on a washing solution consisting of HCl, ultra-pure ethanol, and type I water (1:1:1). This washing process was repeated three times. GO was washed twice with ultra-pure ethanol and type I water (1:1) and once with type I water. Finally, GO was lyophilized and stored at 4 °C until further use (see Figure 1).

The correct synthesis of GO was confirmed by thermogravimetric analysis (TGA), Fourier-transform infrared spectroscopy (FTIR) and Raman spectroscopy. TGA was recorded in a TG analyzer (TA, instruments, New Castle, DE, USA) using a temperature ramp of 25–800 °C with a heating rate of 10 °C/min under a nitrogen atmosphere. FTIR was recorded in an A250 FT-IR (Bruker, Germany) and a spectral range of 4000–400 cm^−1^, and a resolution of 2 cm^−1^. Raman spectra were recorded in an XPlora Raman Horiba confocal (Horiba Scientific, Kyoto, Japan) via laser excitation at 532 nm. Image analysis’ morphology of GO nanosheets was studied using an electron transmission microscope (TEM) Tecnai F30 (FEI Company, Hillsboroc, OR, USA).

### 2.3. Preparation of Gelatin-GO Hydrogels

Sterile milli-Q water was heated up to 40 °C and mixed with gelatin Type A (food grade). The mixture was kept under mechanical stirring at 180 RPM for 30 min. GO solution (1.0 mg/mL) was added at 40 °C and 180 RPM for an additional 5 min. Glutaraldehyde (GTA) solution 25% (*v*/*v*) for crosslinking (PanReac AppliChem, Barcelona, Spain) was added dropwise while stirring at 80 RPM in a water bath at 40 °C for 1 h. Simultaneously, the yeast cells were resuspended in sterile water and carefully poured into the hydrogel solution to reach a final 5.0% (*w*/*v*) concentration. The mixture was cooled down to room temperature and poured into silicone molds food grade (La Orquidea, semi-sphere, Colombia). Finally, the obtained hydrogels were stored at 4 °C until further use. The complete procedure was completed in a laminar flow hood using sterilized materials and equipment (Figure 1).

### 2.4. Preparation of GO Hydrogel Enriched Probiotic Hydrogel

Based on our previous work [68], we chose the concentration level of gelatin and GTA at which the probiotic cells showed the highest cell viability in different experiments. The studied factor was the concentration of GO in the hydrogel. The selected gelatin concentration for this study was 7.5% (*w*/*v*), glutaraldehyde was 3.0% (*w*/*w*), and the probiotic yeast cells were maintained at 5.0% (*w*/*v*). GO was evaluated at 0.1 and 0.2 mg/mL and compared with our previous work where GO was absent (i.e., 0.0 mg/mL) [68]. These low concentrations were chosen to avoid cell viability and swelling issues observed previously at higher concentrations [70,71]. The statistical comparison tests used were analysis of variance, Tukey’s test, and the Q test to discard atypical data.

### 2.5. Survival Rate of Encapsulated Probiotics

Thin cross-sections (about 2 mm thick) of the hydrogel with encapsulated probiotic cells were stained with 50 µL of propidium iodide (frozen stock 1.0 mg/mL, Sigma-Aldrich, Milwaukee, WI, USA), fixed and stained with 50 µL of 4′,6-diamidino-2-phenylindole (DAPI) (frozen stock 1.0 mg/mL, kindly donated by the Department of Biomedical Engineering), and observed under a Confocal Laser Scanning Microscope Olympus FV1000 (40×, 0.6 NA). The live/dead ratio was calculated with the aid of the Fiji-ImageJ^®^ software [72]. These analyses were carried out for the hydrogels after packed bioreactor operation and GI tract treatments. Propidium iodide stains red cells with compromised membranes (i.e., dead cells), while DAPI emits intensive blue when bound to A-T base regions of DNA [73].

### 2.6. Morphological Structure and Beads Conformation

The hydrogels’ surface morphology and presence of encapsulated cells were observed with the JEOL scanning electron microscope (model JSM 6490-LV). The observation was performed on a cooling stage at −15 °C with liquid nitrogen-mediated fracturing to avoid the gel surface’s alterations due to dehydration. Images were obtained at 1000× and 6000× magnification (10 kV) and then processed to determine the average pore size of the hydrogel with the aid of the software ImageJ^®^ [74].

### 2.7. Swelling Percentage Determination

A thin portion of each hydrogel was cut, and its initial weight was recorded. These were then transferred to buffer solutions at different pH values (3.0, 7.0, and 10) and 30 °C. The pH adjustment was performed with hydrochloric acid 37% (*v*/*v*) (PanReac AppliChem, Spain) and sodium hydroxide 1.0 M (solid, PanReac AppliChem, Spain) and verified with a pH meter (Mettler Toledo, Madrid, Spain). The weight change was followed by gravimetry, and the reported value was taken after 24 h. The percentage of swelling was determined according to Equation (1). *W_C_* is the hydration percentage, *W_S_* is the gel’s weight after swelling, and *W_D_* is the initial weight [75].

(1) $$W_{c}=\frac{W_{S}-W_{D}}{W_{D}}\cdot 100,$$

### 2.8. Rheological Response

The rheological analyses were performed in a Discovery Series Hybrid Rheometer-1 (TA Instruments, New Castle, DE, USA), acquiring storage modulus by running an oscillatory frequency scan between 0.62 and 62 rad/s at 1.0% strain and 20 °C. A parallel plate (diameter 20 mm) geometry was used with a fixed gap distance (1.0 mm) between the plates [76]. A sample amount corresponding to 20 mm diameter and 1 mm thickness was used for this characterization (between 200 and 300 mg).

### 2.9. Performance of Encapsulates in a Milliliter Scale Bioreactor

The gelatin-GO encapsulates were tested in a milliliter scale (250 mL), external-loop airlift-bioreactor. The base was manufactured in polylactic acid (PLA), while the body and lid were built from commercially available polypropylene. The external loop and connectors were cast in silicone rubber using 3D-printed molds (Stratasys, Eden Prairie, MN, USA). Details are presented in Supplementary Material (Figure S1). Aseptically, 15 half-sphere hydrogels were placed in the reactor, and the culture medium was then added to reach a 190 mL operation volume. The system was maintained at 30 °C with an aeration supply provided by an air pump (AC9904 RESUN, 8 W) for 72 h.

### 2.10. Thermal Stability Analyses

Thermogravimetric analyses (TGA) were conducted in the range of 20–500 °C to estimate the hydrogels’ thermal stability. About 15–25 mg of the polymeric hydrogel was heated up at a constant rate (10 °C/min) under a controlled atmosphere with 100 mL/min ultra-high purity Nitrogen (UHP). The hydrogels were evaluated by collecting thermograms before and after testing in the milliliter scale bioreactor (see Section 2.9 below for more information). The instrument used was the Q600 Simultaneous TGA/DSC (TA Instruments, New Castle, DE, USA).

### 2.11. Mechanical Resistance Evaluation

Hydrogel’s firmness was evaluated with the aid of a TA.HDplusC Texture Analyzer (Stable Micro Systems, Godalming, UK) before and after the samples were tested in the bioreactor for 72 h and after each GIT simulation treatment. The gels were cut into 20 mm diameter and 10 mm height cylinders. This test measured compression force at a 1.0 mm/s speed and 5.0 mm penetration length using a 35 mm cylindrical probe.

### 2.12. Performance of Encapsulates in the Simulated Gastrointestinal Tract (GIT) Media

A single half-sphere hydrogel was placed in a 250 mL flask with 100 mL of different, consecutive, and sterile solutions simulating saliva, stomach, and small intestine conditions. These solutions were prepared according to our previous work [68]. The treatment began by exposing the hydrogel to the simulated saliva medium for 7 min, then to the stomach medium for 2 h, and finally to the small intestine medium for two more hours. The process was performed at 37 °C and 150 RPM.

## 3. Results

### 3.1. Graphene Oxide Synthesis and Characterization

**Figure 2:**
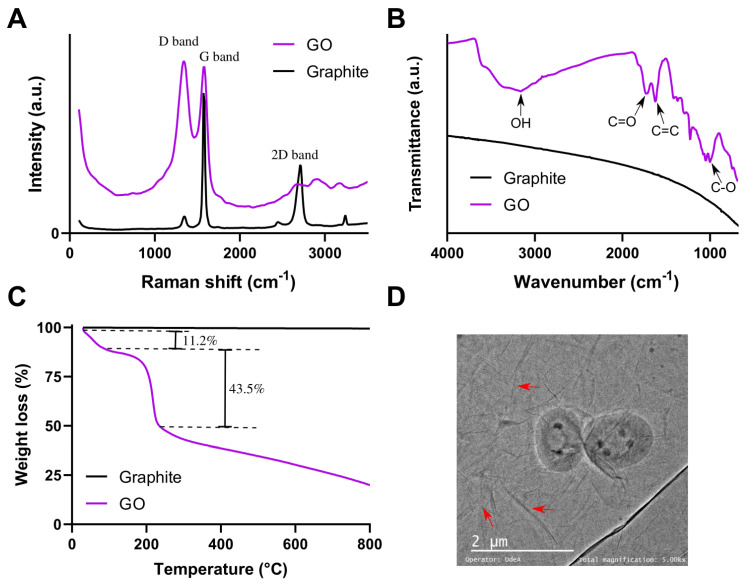
(**A**) Raman spectra of GO and graphite. (**B**) FTIR spectra of GO and graphite. (**C**) TGA of GO and graphite. (**D**) TEM image of GO flakes: red arrows point to folds on GO flakes.

GO was characterized by Raman spectroscopy, FTIR and TGA to confirm the synthesis and the oxidation level. Figure 2A shows the Raman spectra of GO and graphite as a reference. The characteristic D (1343.07 cm^−1^) and G (1583.41 cm^−1^) bands are observed, confirming the lattice distortions [69]. The D band with a high intensity corresponds to the disruption of Sp^2^ bonds of the carbon, related to the presence of oxidative functional groups on the surface of GO [77]. FTIR spectrum of GO (see Figure 2B) exhibits several peaks related to the oxidative functional groups, namely O-H stretching vibrations at 3420 cm^−1^, C=O stretching vibrations in the range of 1720–1740 cm^−1^, C=C from unoxidized sp2 CC bonds in the range of 1590–1620 cm^−1^ and C-O stretching vibrations at 1250 cm^−1^ [69,77]. Figure 2C shows the thermograms of GO and graphite. The GO thermogram shows three prominent weight losses. The first weight loss (11.2%) at 100–150 °C corresponds to dehydration, a second loss (43.5%) between 150 and 200 °C is from most labile functional groups, and the last mass loss, between 200 and 800 °C can be attributed to the removal of stable oxygen functionalities [69,77]. Finally, Figure 2D shows a TEM image of GO. Red arrows indicate folds in GO flakes. In terms of yield in the synthesis protocol, we started from 0.75 g of graphite flakes to obtain 1.45 mg of GO.

### 3.2. Morphological Structure of Hydrogels and Encapsulated Cells

**Figure 3:**
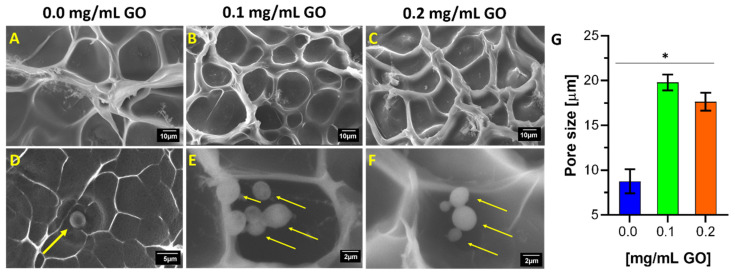
Surface morphology described by scanning electron microscope (SEM) micrographs of cross-linked and uncross-linked hydrogels in the presence and absence of yeast cells. First row is for 7.5% (*w*/*v*) gelatin and 3.0% (*w*/*w*) GTA concentration and varying GO concentrations from 0.0% (**A**), 0.1% (**B**) to 0.2% (*w*/*w*) (**C**). Second row (**D**–**F**) is for the same formulation but at a higher magnification. Images also include yellow arrows to point to *K. lactis* cells compartmentalized into the gel pores. Finally, the average pore size is shown for the treatments (**G**). *, *p* < 0.05.

The collected images shown in Figure 3A–C confirmed that an increase in GO concentration leads to an increase in the average pore size of the gel. This is expected, considering that the encapsulates were prepared with a double cross-linking procedure compared to a simple one in our previous work (see Figure 1). In consequence, the obtained polymeric matrix exhibits higher compactness and cohesiveness. Moreover, this difference in pore size is statistically significant and particularly noticeable concerning the formulation in the absence of GO. This considerable variation may seem counterintuitive at first. Still, it can be most likely explained by the fact that GTA-mediated chain–chain and chain–GO cross-linking compete during the process, and therefore, the amount of GO needs to be increased considerably to achieve binding to more chains. In other words, it is very likely that a small fraction of the hydrogel is doubly cross-linked (chain–chain and chain–GO) while the vast majority of chains remain either singly cross-linked or unchanged. Similar results have been observed for GO hydrogels with rough and wrinkled microstructures [78,79]. This leads to both more uneven and higher porosity than a gel only cross-linked with GTA. However, no GO aggregates were evidenced, so the dispersion in the hydrogel matrix was homogeneous [80]. Additionally, this may suggest a strong interfacial adhesion interaction between the GO nanocomposite and the gelatin polymeric chain [62,81].

The microstructure of double cross-linked hydrogels with GO resembles a honeycomb. It also exhibits pores in the size range of several micrometers [82,83]. These unique microstructural features (not previously seen in the absence of GO) can also be explained by the GO sheets’ flexibility to build the three-dimensional mesh and interact with the chains of the macrostructure polymeric material [82]. Figure 3G confirms that the average pore size is strongly dependent on GO and its concentration.

The pore size of surface morphologies confirmed the effectiveness of the cross-linking via GTA-GO. Additionally, a lower variability is observed than in the formulation and experimental design in the absence of GO. The micrographs allowed direct visualization of cells fixed on the hydrogel surface. In comparison with the formulation in the absence of GO (Figure 3D), Figure 3E,F shows that the larger pore sizes induced by GO not only act as microchambers to house the cells but allow a higher cell proliferation per pore. The micrographs also suggest a decrease in the average cell size related to the sample’s dehydration in the liquid nitrogen treatment required before imaging.

### 3.3. Swelling Degree of Nanocomposite Hydrogels

**Figure 4:**
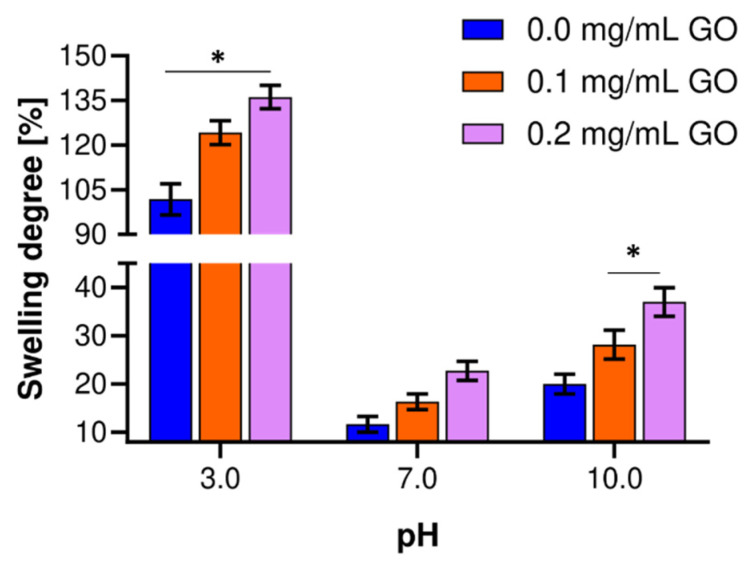
Swelling behavior of hydrogels prepared with varying levels of GO and different pH values. The experiments were conducted in an aqueous solution and 30 °C. *, *p* < 0.05.

Hydrogels were allowed to swell in buffer solutions at 30 °C. Figure 4 shows the degree of swelling for the different GO concentrations. When the GO concentration increases in the hydrogel for all the pH values studied, the swelling degree also increases, comparable to previous studies [84]. Noticeably, at pH 3.0, the capacity of the hydrogels to retain water within their structure without deformation is significantly higher compared with pH 7.0 and 10.0. Additionally, at this pH, the swelling differences are statistically significant between treatments, with a maximum of about 130% for the highest GO concentration. This exceedingly high swelling capacity may be related to the protonation of amino groups in uncross-linked gelatin chains, which increases electrostatic interactions and chain–chain repulsion, and therefore the ability of the hydrogel structure to remain distended in the presence of water [82]. At pH 10.0, the highest GO concentration allowed about 40% swelling, statistically different from the other treatments. Even though this swelling level was above that of pH 7.0, only a fraction of that was obtained at pH 10. At pH 7.0, there was a limited swelling capacity, most likely because the isoelectric point of the material is near the evaluated buffer solution. At pH 10.0, gelatin amino groups deprotonated and most likely destabilized the cross-linking structure of hydrogel, detrimentally reducing the swelling capacity [85]. However, this acceptable level of swelling might be attributed to negatively charged groups within the hydrogel, which undergo repulsive interaction and promote matrix expansion to include more water [86], and the GO incorporated in the hydrogel. The nanocomposite improved the cross-linking density, and its hydrophilicity enabled better matrix water retention performance, which is likely responsible for inducing a microstructural reinforcement [85,87]. The swelling ranges between 10 and 20% at neutral pH without any statistically significant differences between treatments. This deficient swelling level can be explained by the absence of interactions with free ions, which, in turn, leads to facile dissolution [86]. Compared with hydrogels without GO, the presence of the nanomaterial leads to superior swelling capacity, possibly due to the prevalence of heavily cross-linked patches where very stable covalent bonds provide resilience to the increased tension induced by the incorporated water molecules, which, in turn, penetrate the matrix easily by the higher permeability resulting from a larger pore size distribution [61,76].

This notion is further supported by the high correlation observed between low cross-linking levels (0.0 mg/mL GO) and reduced swelling degrees and pore sizes. In that case, the smaller pore sizes are likely to impose a physical impediment for the water molecules to reach the hydrogel matrix’s interior [84,86,87]. Additionally, an important observation is that after 24 h in aqueous solution, none of the GO hydrogels showed structural stability failure or observable changes in their macrostructure. Putting all together, these findings strongly suggest that GO hydrogels are likely to offer better tolerance to the transit along the GI tract compared with the pristine ones. According to the results, hydrogels have a swelling behavior sensitive to pH, which is of great interest, mainly for probiotics’ effective delivery.

### 3.4. Rheological Response of Hydrogels Nanocomposites

**Figure 5:**
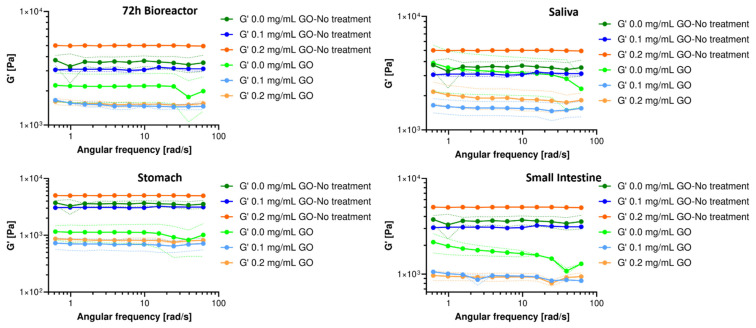
Storage (G’) modulus for hydrogels after exposure to milli-bioreactor operation for 72 h and human gastrointestinal tract (GIT) simulated media and the comparison with a hydrogel in the absence of each treatment.

The oscillatory frequency tests reveal that the storage modulus (G’) is higher than the loss modulus (G”) in the studied range both before and after each treatment. However, just G’ is presented in Figure 5. This shows that the GO-modified hydrogels exhibited a solid-like behavior and good resistance to external forces even when subjected to high oscillatory stress values [88]. This also suggests that the elastic response dominates the nanocomposite hydrogels, demonstrating their strong stability [62,89]. After serving as carriers of the probiotic cells in the packed bioreactor, it was observed that the magnitude of their G’ decreased significantly after treatment for all the evaluated GO concentrations. However, it remains in the same order of magnitude and with a trend that likely indicates that G’ is independent of the angular frequency. The constant slope and independence of the angular frequency observed for the G’ curves suggest a heavily cross-linked matrix, as would be expected for increasing GO concentrations.

The hydrogels’ rheological response after exposure to each simulated GIT medium is also shown in Figure 5. After passing through the simulated saliva medium, a significant decrease in G’ was observed for both GO treatments. Although the exposure was for a short time, the enzymatic activity and pH of the medium generated noticeable destabilization of the hydrogel, maintaining the trend throughout the evaluated frequency range. After exposure to the simulated stomach medium, the hydrogels showed an evident and significant decrease in G’ for all formulations, becoming one order of magnitude lower for the GO treatments. The proteolytic and enzymatic activity, the aggressive pH, and the exposure time most likely led to a decrease in the hydrogel’s structural stability, but it remains as a weak solid. This provides further evidence for a considerable chain rearrangement, changes in orientation or chain mobility, induced disorder at the molecular level, and unstable cross-linking [90,91]. This marked alteration in the 3D structural organization confirms the detrimental impact of low pH conditions on the nanocomposite’s macroscopic stability.

Finally, after exposure to the small intestine’s simulated medium, the hydrogel nanocomposites showed an apparent stabilization in the magnitude of G’, which most likely indicates some level of chain rearrangement to achieve the three-dimensional bonding required for a solid-like material [84,87,88,89,90,91]. Importantly, taken together, these results suggest that during the pass through the GI tract, the material will continue to exhibit a solid-like rheological response, which is critical to assure that a large population of probiotics effectively reaches the site of action. This indicates that the nanocomposite hydrogel most likely increases its elasticity and becomes mechanically stronger, favoring applications for the controlled delivery of probiotics and other bioactive molecules. Moreover, this behavior might be exploitable to enable other biological and biomedical applications, including regenerative therapies, cancer therapy, bioadhesives, wastewater treatment, packaging, and coatings [60,67,84,88,91].

### 3.5. Mechanical Resistance Evaluation

**Figure 6:**
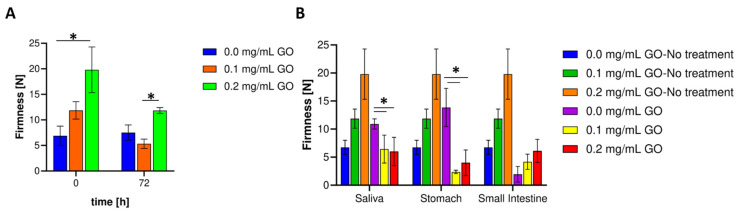
Evaluation of hydrogels’ mechanical response. (**A**) Firmness for chemically cross-linked hydrogels before and after the milli-bioreactor operation. (**B**) Hydrogel firmness after exposure to gastrointestinal tract simulated media at different pH values. *, *p* < 0.05.

Figure 6A shows the firmness of the nanocomposite hydrogels before and after operation in the bioreactor for 72 h compared to the pristine hydrogel. In contrast with the pristine material, as the amount of GO in the composite increases, the firmness increases [92]. This is most likely because the obtained polymeric matrices exhibit chain–chain and chain–GO cross-linking, resulting in microstructural features that provide greater resistance to deformation by an external force. In Table 1, after completing 72 h in the bioreactor under continuous aeration, the decrease in firmness concerning the beginning is significant for the GO treatments, albeit remained at the same level or slightly above the pristine materials. This may be because, despite GO’s exceptional mechanical resistance, gelatin starts to degrade faster at the rich GO clusters, most likely due to mechanical stress propagation at such sites. This appears to be exacerbated when GO has sufficient space for displacement, i.e., at the lower concentration of 0.1 mg/mL [93,94,95,96].

Figure 6B shows that after treatment with the simulated saliva medium, the nanocomposite hydrogels’ firmness decreases by about 45.68% (0.1 mg/mL) and 69.63% (0.2 mg/mL) with respect to the initial value. Additionally, after saliva simulation, the hydrogel without GO increased 62% concerning the initial pristine material without any treatment. A similar result was found for the stomach simulation where the reductions approached 80% (0.1 mg/mL) and 79.8% (0.2 mg/mL), respectively. This provides further evidence for the notion that there may be substantial hydrogel’s structural rearrangements, which, in turn, are most likely triggered by changes in environmental conditions such as pH, salt composition, enzymatic activity, and ionic exchange. Finally, after exposure to the small intestine’s simulated environment, the nanocomposites’ firmness achieves an apparent stabilization as no significant differences were detected with respect to the values obtained after passage through the simulated stomach medium. This result is encouraging because it proves that the prepared nanocomposites can overcome the hostile environment of the stomach to arrive at the intestine with sufficient integrity to maintain largely intact probiotic cells. Additionally, once they reach this lower portion of the GI tract, a pH close to neutrality would favor the progressive disintegration of the hydrogel.

### 3.6. Thermal Resistance Evaluation

**Figure 7:**
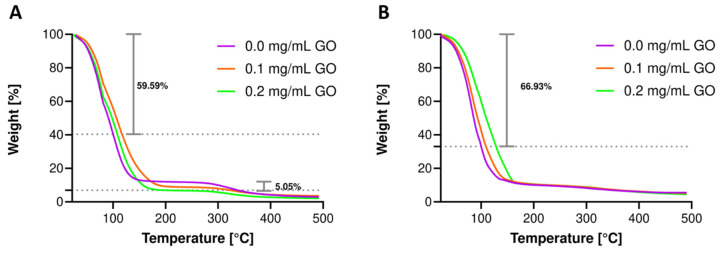
The average weight (%) loss at 100 °C is indicated on each plot. Thermograms for hydrogels with 7.5% (*w*/*v*) gelatin concentration and GTA concentration of 3.0% (*w*/*w*). Thermal degradation for hydrogels without any treatment (**A**) and after 72 h milli-bioreactor operation (**B**).

Thermogravimetric analyses were conducted in the range of room temperature to 500 °C to estimate the nanocomposite hydrogels’ thermal stability before and after bioreactor operation. As observed in our previous work, Figure 7A shows a pronounced weight drop at 100 °C averaging 59.59% of the initial weight for pristine materials and nanocomposites, directly related to the water’s evaporation occluded in the hydrogel. The hydrogel nanocomposite remained largely unaltered, as evidenced by the high resistance to degradation up to 300 °C. After this temperature, GO has been severely degraded, leaving some residual carbon, and hence, the weight difference of 5.05% with respect to the pristine hydrogel is evident, and thermograms show convergence between treatments for final gelatin degradation. Additional thermal resistance might be achieved by incorporating GO into the hydrogel formulation above the levels explored here. Figure 7B shows the thermal degradation after 72 h in the bioreactor, where the initial weight loss is even more prominent, approaching 66.93%. After evaporation, the thermograms stabilize and converge towards 8% weight after 350 °C, reducing the weight loss obtained before the treatment. This confirms the strong interaction of GO with gelatin, altering its degradation, extending it to broader temperature ranges. With respect to the treatments, it can be inferred that gelatin degradation occurs during the operation of the encapsulates in the bioreactor. This can be explained by the toughening of the matrix by interactions with molecules present in the fermentative medium that continuously diffuse in and out of the system. This includes sugars, salts, and ions or metabolites produced and secreted extracellularly by the probiotic strain, leading to reduced polymer chain mobility [94,95]. Moreover, some of these compounds might end up occluded within the hydrogel, which would explain the reduced loss of weight towards the end of the experiment. The result agrees well with the measured firmness obtained after this treatment since approximately the same level was achieved for the nanocomposite hydrogels and the pristine ones.

### 3.7. Cell Viability Assays

**Figure 8:**
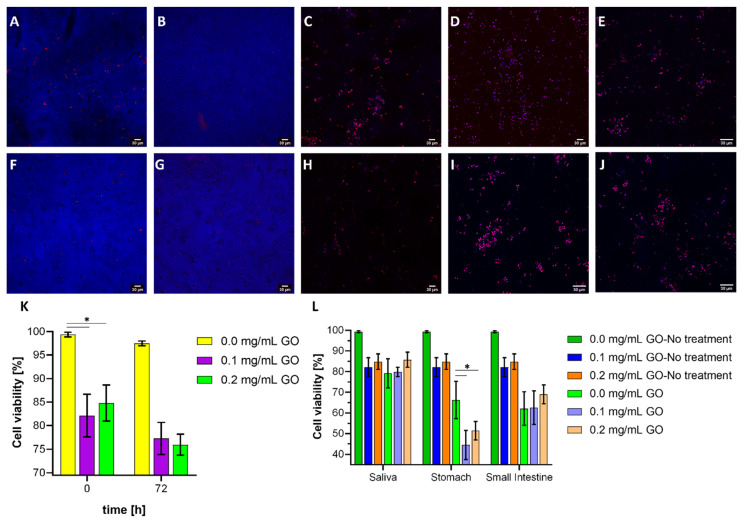
Confocal microscopy images. Dead cells are shown in red color while live cells in blue. Scale bar corresponds to 30 µm. Live/dead *K. lactis* cells in the encapsulates made with 0.1 mg/mL GO at the beginning (**A**), after 72 h of bioreactor operation (**B**), after exposure to simulated saliva medium (**C**), after exposure to simulated stomach medium (**D**), and after exposure to the simulated small intestine medium (**E**). Live/dead *K. lactis* cells in the encapsulates made with 0.2 mg/mL GO at the beginning (**F**), after 72 h of bioreactor operation (**G**), after exposure to simulated saliva medium (**H**), after exposure to simulated stomach medium (**I**), and after exposure to the simulated small intestine medium (**J**). Yeast probiotic cell survival rate for encapsulates before and after bioreactor operation (**K**) and after treatment with each of the gastrointestinal tract simulated media (**L**). *, *p* < 0.05.

According to the images in Figure 8A,B,F,G, the probiotic strain’s cell viability is compromised by GO in the hydrogel formulation. This decrease is significant compared to the pristine hydrogel both before and after operation in the bioreactor. However, no differences in cell viability over time were identified between the two GO levels studied (Figure 8K). Nevertheless, a minimum survival rate of 73% is assured by the end of 72 h. Although a larger pore size was observed for the nanocomposite hydrogels, which would facilitate the proliferation and diffusion of nutrients from the medium to its interior, their lower mechanical performance may be detrimental for cell fixation and subsequent proliferation. Notably, the nanocomposite hydrogel cell proliferation allowed survival rate recovery [97,98,99,100]. Further analyses need to be planned to investigate in detail the antimicrobial activity of GO (free and cross-linked) in the range of studied concentrations. Moreover, it will be valuable to evaluate possible undesirable effects when transiting in the organism by in vivo assays or by using simplified models that rely on human stem cells, as discussed previously in the work of Hasda [92]. However, recent evidence suggests induction of mutagenesis, apoptosis, and proliferation inhibition in human cell lines after prolonged exposure to GO [101].

The images in Figure 8C,H show no significant differences in cell viability after exposure to the simulated saliva medium, mainly because it occurred during a short period where the hydrogels’ mechanical stability and macroscopic integrity were maintained. After exposure to the simulated stomach medium, cell viability drops significantly to 44.56% (0.1 mg/mL GO) and 51.40% (0.2 mg/mL GO), which was attributed to the extreme pH and loss of structural integrity that can cause cells to be released early into the medium (Figure 8L). The subsequent exposure to the simulated small intestine ends medium occurred at a neutral pH, which favored the proliferation of viable cells and, consequently, higher viability, ending at a minimum of about 54%. These results are certainly encouraging as they demonstrate that the GO-modified nanocomposite hydrogels can overcome the conditions along the GI tract to effectively carry highly viable probiotic microorganisms to the intestine for further therapeutic action.

## 4. Conclusions

Despite the growing interest in probiotics as an integral part of more functional food products, a significant challenge that remains largely unsolved is ensuring that, once administered, they transit the GI tract and reach the site of action with high viability. Here, we aimed to improve the stability of probiotic encapsulates by forming nanocomposite hydrogels of gelatin cross-linked with GO. SEM micrographs confirmed larger pore sizes for the nanocomposites, which is helpful for cell entrapment and proliferation. Their swelling capacity showed strong pH dependence with the highest resistance and distensibility at low pH values. The rheological and mechanical characterization showed sufficient stability after undergoing the treatments of interest. No significant changes were observed for the thermal stability of the composites, as evidenced by very similar thermograms with respect to those in the absence of GO. Finally, the nanocomposite allowed the probiotic cell viability to reach 73% and 54% after 72 h under bioreactor operation and sequential exposure to simulated GIT media, respectively. We are confident that these results pave the way for the possibility of formulating more robust nutraceuticals and functional food products based on probiotics encapsulation. Finally, we believe that the development can also be exploited for the controlled delivery of drugs and bioactive molecules with limited absorption through the intestinal lumen.

## Figures and Tables

**Table 1:** Arithmetic mean and standard deviation of the hydrogels’ firmness for the different treatments studied.

	0.0 mg/mL GO		0.1 mg/mL GO		0.2 mg/mL GO	
Treatment	Mean	SD	Mean	SD	Mean	SD
None	6.880	1.907	11.863	1.707	19.807	4.478
72 h bioreactor	7.516	1.495	5.321	0.923	11.834	0.572
Saliva	10.913	0.902	6.444	2.486	6.014	2.524
Stomach	13.853	3.406	2.369	0.300	4.000	2.286
Small intestine	1.950	1.393	4.174	1.355	6.105	2.072

## Supplementary Materials

The following are available online at https://www.mdpi.com/article/10.3390/biom11070922/s1, Figure S1. Packed-bed bioreactor designed and 3D-printed for this study. (A) 3D model. (B) Isometric view. (C) Half-sphere GO nanocomposite hydrogels used for packing bioreactor. (D) Gel sample without GO.

## References

[1] Shimoda A., Yamamoto Y., Sawada S.I., Akiyoshi K. (2012). Biodegradable nanogel-integrated hydrogels for sustained protein delivery. Macromol. Res..

[2] Martínez-Martínez M., Rodríguez-Berna G., Bermejo M., Gonzalez-Alvarez I., Gonzalez-Alvarez M., Merino V. (2019). Covalently crosslinked organophosphorous derivatives-chitosan hydrogel as a drug delivery system for oral administration of camptothecin. Eur. J. Pharm. Biopharm..

[3] Gulen B., Demircivi P. (2020). Synthesis and characterization of montmorillonite/ciprofloxacin/TiO2 porous structure for controlled drug release of ciprofloxacin tablet with oral administration. Appl. Clay Sci..

[4] Shah A.V., Desai H.H., Thool P., Dalrymple D., Serajuddin A.T.M. (2018). Development of self-microemulsifying drug delivery system for oral delivery of poorly water-soluble nutraceuticals. Drug Dev. Ind. Pharm..

[5] Wu S., Bin W., Tu B., Li X., Wang W., Liao S., Sun C. (2019). A Delivery System for Oral Administration of Proteins/Peptides Through Bile Acid Transport Channels. J. Pharm. Sci..

[6] Horigome A., Hisata K., Odamaki T., Iwabuchi N. (2021). Colonization of Supplemented Bifidobacterium breve M-16V in Low Birth Weight Infants and Its Effects on Their Gut Microbiota Weeks. Front. Microbiol..

[7] Cazorla S.I., Maldonado-Galdeano C., Weill R., De Paula J., Perdigón G.D.V. (2018). Oral administration of probiotics increases Paneth cells and intestinal antimicrobial activity. Front. Microbiol..

[8] Secher T., Kassem S., Benamar M., Bernard I., Boury M., Barreau F., Oswald E., Saoudi A. (2017). Oral administration of the probiotic strain Escherichia coli Nissle 1917 reduces susceptibility to neuroinflammation and repairs experimental autoimmune encephalomyelitis-induced intestinal barrier dysfunction. Front. Immunol..

[9] Aindelis G., Tiptiri-kourpeti A., Lampri E., Spyridopoulou K., Lamprianidou E., Kotsianidis I., Ypsilantis P., Pappa A., Chlichlia K. (2020). Immune Responses Raised in an Experimental Colon. Cancers.

[10] Nikam P.S., Kingston J.J., Belagal Motatis A.K. (2021). Oral co-administration of bivalent protein r-BL with U-Omp19 elicits mucosal immune responses and reduces S. Typhimurium shedding in BALB/c mice. Immunol. Lett..

[11] Li J., Chang A.K., Li Y., Tao X., Liu W., Su W., Li Z., Liang X. (2021). Screening and tissue distribution of protein tyrosine phosphatase 1B inhibitors in mice following oral administration of Garcinia mangostana L. ethanolic extract. Food Chem..

[12] Han S., Lu Y., Xie J., Fei Y., Zheng G., Wang Z., Liu J., Lv L., Ling Z., Berglund B. (2021). Probiotic Gastrointestinal Transit and Colonization After Oral Administration: A Long Journey. Front. Cell. Infect. Microbiol..

[13] Gleeson J.P. (2017). Diet, food components and the intestinal barrier. Nutr. Bull..

[14] Wright L., Barnes T.J., Prestidge C.A. (2020). Oral delivery of protein-based therapeutics: Gastroprotective strategies, physiological barriers and in vitro permeability prediction. Int. J. Pharm..

[15] Raddatz G.C., de Menezes C.R. (2021). Microencapsulation and co-encapsulation of bioactive compounds for application in food: Challenges and perspectives. Cienc. Rural.

[16] Misra S., Pandey P., Mishra H.N. (2021). Novel approaches for co-encapsulation of probiotic bacteria with bioactive compounds, their health benefits and functional food product development: A review. Trends Food Sci. Technol..

[17] Šeregelj V., Ćetković G., Čanadanović-Brunet J., Tumbas Šaponjac V., Vulić J., Stajčić S. (2020). Encapsulation and degradation kinetics of bioactive compounds from sweet potato peel during storage. Food Technol. Biotechnol..

[18] Abdul Mudalip S.K., Khatiman M.N., Hashim N.A., Che Man R., Arshad Z.I.M. (2021). A short review on encapsulation of bioactive compounds using different drying techniques. Mater. Today Proc..

[19] Zhang Q., Zhou Y., Yue W., Qin W., Dong H., Vasanthan T. (2021). Nanostructures of protein-polysaccharide complexes or conjugates for encapsulation of bioactive compounds. Trends Food Sci. Technol..

[20] Khayambashi P., Iyer J., Pillai S., Upadhyay A., Zhang Y., Tran S.D. (2021). Hydrogel encapsulation of mesenchymal stem cells and their derived exosomes for tissue engineering. Int. J. Mol. Sci..

[21] Gómez-Mascaraque L.G., Martínez-Sanz M., Fabra M.J., López-Rubio A. (2019). Development of gelatin-coated ι-carrageenan hydrogel capsules by electric field-aided extrusion. Impact of phenolic compounds on their performance. Food Hydrocoll..

[22] Demisli S., Mitsou E., Pletsa V., Xenakis A., Papadimitriou V. (2020). Development and study of nanoemulsions and nanoemulsion-based hydrogels for the encapsulation of lipophilic compounds. Nanomaterials.

[23] Wang B., Wang S., Zhang Q., Deng Y., Li X., Peng L., Zuo X., Piao M., Kuang X., Sheng S. (2019). Recent advances in polymer-based drug delivery systems for local anesthetics. Acta Biomater..

[24] Bai S., Jia D., Ma X., Liang M., Xue P., Kang Y., Xu Z. (2021). Cylindrical polymer brushes-anisotropic unimolecular micelle drug delivery system for enhancing the effectiveness of chemotherapy. Bioact. Mater..

[25] Kretzmann J.A., Luther D.C., Evans C.W., Jeon T., Jerome W., Gopalakrishnan S., Lee Y.-W., Norret M., Iyer K.S., Rotello V.M. (2021). Regulation of Proteins to the Cytosol Using Delivery Systems with Engineered Polymer Architecture. J. Am. Chem. Soc..

[26] Bernkop-Schnurch A., Malkawi A., Jalil A., Nazir I., Matuszczak B., Kennedy R. (2020). Self-emulsifying drug delivery systems: Hydrophobic drug polymer complexes provide a sustained release in vitro. Mol. Pharm..

[27] Mdlovu N.V., Lin K.S., Chen Y., Juang R.S., Chang T.W., Mdlovu N.B. (2019). Formulation and characterization of multifunctional polymer modified-iron oxide magnetic nanocarrier for doxorubicin delivery. J. Taiwan Inst. Chem. Eng..

[28] Pinteala M., Abadie M.J.M., Rusu R.D. (2020). Smart supra- and macro-molecular tools for biomedical applications. Materials.

[29] Municoy S., Álvarez Echazú M.I., Antezana P.E., Galdopórpora J.M., Olivetti C., Mebert A.M., Foglia M.L., Tuttolomondo M.V., Alvarez G.S., Hardy J.G. (2020). Stimuli-responsive materials for tissue engineering and drug delivery. Int. J. Mol. Sci..

[30] Reza Saboktakin M., Tabatabaei R.M. (2015). Supramolecular hydrogels as drug delivery systems. Int. J. Biol. Macromol..

[31] Ebara M., Kotsuchibashi Y., Narain R. (2014). Smart Biomaterials.

[32] Liarou E., Varlas S., Skoulas D., Tsimblouli C., Sereti E., Dimas K., Iatrou H. (2018). Smart polymersomes and hydrogels from polypeptide-based polymer systems through α-amino acid N-carboxyanhydride ring-opening polymerization. From chemistry to biomedical applications. Prog. Polym. Sci..

[33] Zhang H., Niu C., Zhang Y., Wang X., Yang B. (2020). A mechanically strong polyvinyl alcohol/poly(2-(N,N′-dimethyl amino) ethyl methacrylate)-poly (acrylic acid) hydrogel with pH-responsiveness. Colloid Polym. Sci..

[34] Puertas-Bartolomé M., Benito-Garzón L., Fung S., Kohn J., Vázquez-Lasa B., San Román J. (2019). Bioadhesive functional hydrogels: Controlled release of catechol species with antioxidant and antiinflammatory behavior. Mater. Sci. Eng. C.

[35] Vafaei A., Rahbarghazi R., Kharaziha M., Avval N.A., Rezabakhsh A., Karimipour M. (2021). Polycaprolactone fumarate acts as an artificial neural network to promote the biological behavior of neural stem cells. J. Biomed. Mater. Res. Part B Appl. Biomater..

[36] Puentes P.R., Henao M.C., Torres C.E., Gómez S.C., Gómez L.A., Burgos J.C., Arbeláez P., Osma J.F., Muñoz-Camargo C., Reyes L.H. (2020). Design, screening, and testing of non-rational peptide libraries with antimicrobial activity: In silico and experimental approaches. Antibiotics.

[37] Giubilini A. (2019). Antibiotic resistance as a tragedy of the commons: An ethical argument for a tax on antibiotic use in humans. Bioethics.

[38] Vargason A.M., Anselmo A.C. (2020). Evaluation of Surface Modified Live Biotherapeutic Products for Oral Delivery. ACS Biomater. Sci. Eng..

[39] James A., Wang Y. (2019). Characterization, health benefits and applications of fruits and vegetable probiotics. CYTA J. Food.

[40] Hammam A.R.A., Ahmed M.S.I. (2019). Technological aspects, health benefits, and sensory properties of probiotic cheese. SN Appl. Sci..

[41] Bamgbose T., Anvikar A.R., Alberdi P., Abdullahi I.O., Inabo H.I., Bello M., Cabezas-Cruz A., de la Fuente J. (2021). Functional Food for the Stimulation of the Immune System Against Malaria. Probiotics Antimicrob. Proteins.

[42] Sabir F., Qindeel M., Zeeshan M., Ain Q.U., Rahdar A. (2021). Onco-Receptors Targeting in Lung Cancer via Application of Surface-Modified and Hybrid Nanoparticles: A Cross-Disciplinary Review. Processes.

[43] Dash B.S., Jose G., Lu Y.J., Chen J.P. (2021). Functionalized reduced graphene oxide as a versatile tool for cancer therapy. Int. J. Mol. Sci..

[44] Yu H., Park J.Y., Kwon C.W., Hong S.C., Park K.M., Chang P.S. (2018). An overview of nanotechnology in food science: Preparative methods, practical applications, and safety. J. Chem..

[45] Lattuada E., Leo M., Caprara D., Salvatori L., Stoppacciaro A., Sciortino F., Filetici P. (2020). DNA-GEL, Novel Nanomaterial for Biomedical Applications and Delivery of Bioactive Molecules. Front. Pharmacol..

[46] Amini S.M. (2019). Preparation of antimicrobial metallic nanoparticles with bioactive compounds. Mater. Sci. Eng. C.

[47] Mladenovi M., Saoud M., Pergal M.V. (2021). pH-Responsive Release of Ruthenium Metallotherapeutics from Mesoporous Silica-Based Nanocarriers. Pharmaceutics.

[48] Spirescu V.A., Chircov C., Grumezescu A.M., Andronescu E. (2021). Polymeric Nanoparticles for Antimicrobial Therapies: An Up-to-Date Overview. Polymers.

[49] Ghawanmeh A.A., Ali G.A.M., Algarni H., Sarkar S.M., Chong K.F. (2019). Graphene oxide-based hydrogels as a nanocarrier for anticancer drug delivery. Nano Res..

[50] Shan S., Jia S., Lawson T., Yan L., Lin M., Liu Y. (2019). The use of TAT peptide-functionalized graphene as a highly nuclear-targeting carrier system for suppression of choroidal melanoma. Int. J. Mol. Sci..

[51] Li Y., Zheng X., Chu Q. (2021). Bio-based nanomaterials for cancer therapy. Nano Today.

[52] Andretto V., Rosso A., Briançon S., Lollo G. (2021). Nanocomposite systems for precise oral delivery of drugs and biologics. Drug Deliv. Transl. Res..

[53] Rabiee N., Bagherzadeh M., Ghadiri A.M., Fatahi Y. (2021). Bio-multifunctional noncovalent porphyrin functionalized carbon—based nanocomposite. Sci. Rep..

[54] Gholamali I., Yadollahi M. (2021). Bio-nanocomposite Polymer Hydrogels Containing Nanoparticles for Drug Delivery: A Review. Regen. Eng. Transl. Med..

[55] Ghibaudo F., Gerbino E., Copello G.J., Campo Dall’ Orto V., Gómez-Zavaglia A. (2019). Pectin-decorated magnetite nanoparticles as both iron delivery systems and protective matrices for probiotic bacteria. Colloids Surf. B Biointerfaces.

[56] Reddy S., He L., Ramakrishana S., Luo H. (2019). Graphene nanomaterials for regulating stem cell fate in neurogenesis and their biocompatibility. Curr. Opin. Biomed. Eng..

[57] Shi H., Liu W., Xie Y., Yang M., Liu C., Zhang F., Wang S., Liang L., Pi K. (2021). Synthesis of carboxymethyl chitosan-functionalized graphene nanomaterial for anticorrosive reinforcement of waterborne epoxy coating. Carbohydr. Polym..

[58] Talesara V., Garman P.D., Lee J.L., Lu W. (2020). Thermal management of high-power switching transistors using thick CVD-Grown graphene nanomaterial. IEEE Trans. Power Electron..

[59] Phan L.M.T., Vo T.A.T., Hoang T.X., Cho S. (2021). Graphene integrated hydrogels based biomaterials in photothermal biomedicine. Nanomaterials.

[60] Bellet P., Gasparotto M., Pressi S., Fortunato A., Scapin G., Mba M., Menna E., Filippini F. (2021). Graphene-based scaffolds for regenerative medicine. Nanomaterials.

[61] Liu Y., Lyu Y., Hu Y., An J., Chen R., Chen M., Du J., Hou C. (2021). Novel Graphene Oxide Nanohybrid Doped Methacrylic Acid Hydrogels for Enhanced Swelling Capability and Cationic Adsorbability. Polymers.

[62] Zhao X., Zou X., Ye L. (2017). Controlled pH- and glucose-responsive drug release behavior of cationic chitosan based nano-composite hydrogels by using graphene oxide as drug nanocarrier. J. Ind. Eng. Chem..

[63] More M.P., Chitalkar R.V., Bhadane M.S., Dhole S.D., Patil A.G., Patil P.O., Deshmukh P.K. (2019). Development of graphene-drug nanoparticle based supramolecular self assembled pH sensitive hydrogel as potential carrier for targeting MDR tuberculosis. Mater. Technol..

[64] Aderibigbe B.A., Owonubi S.J., Jayaramudu J., Sadiku E.R., Ray S.S. (2014). Targeted drug delivery potential of hydrogel biocomposites containing partially and thermally reduced graphene oxide and natural polymers prepared via green process. Colloid Polym. Sci..

[65] Wang X., Guo W., Li L., Yu F., Li J., Liu L., Fang B., Xia L. (2020). Photothermally triggered biomimetic drug delivery of Teriparatide via reduced graphene oxide loaded chitosan hydrogel for osteoporotic bone regeneration. Chem. Eng. J..

[66] Lu Y.J., Lan Y.H., Chuang C.C., Lu W.T., Chan L.Y., Hsu P.W., Chen J.P. (2020). Injectable thermo-sensitive chitosan hydrogel containing CPT-11-loaded EGFR-targeted graphene oxide and SLP2 shRNA for localized drug/gene delivery in glioblastoma therapy. Int. J. Mol. Sci..

[67] Ali L., Tanzil S., Rehman U., Khan M. (2019). Synthesis of graphene oxide doped poly(2-acrylamido-2-methyl propane sulfonic acid) [GO@p(AMPS)] composite hydrogel with pseudo-plastic thixotropic behavior. Polym. Bull..

[68] Patarroyo J.L., Florez-Rojas J.S., Pradilla D., Valderrama-Rincón J.D., Cruz J.C., Reyes L.H. (2020). Formulation and characterization of gelatin-based hydrogels for the encapsulation of kluyveromyces lactis-Applications in packed-bed reactors and probiotics delivery in humans. Polymers.

[69] Marcano D.C., Kosynkin D.V., Berlin J.M., Sinitskii A., Sun Z., Slesarev A., Alemany L.B., Lu W., Tour J.M. (2010). Improved synthesis of graphene oxide. ACS Nano.

[70] Huang Y., Zeng M., Ren J., Wang J., Fan L., Xu Q. (2012). Preparation and swelling properties of graphene oxide/poly(acrylic acid-co-acrylamide) super-absorbent hydrogel nanocomposites. Colloids Surf. A Physicochem. Eng. Asp..

[71] Yan X., Yang J., Chen F., Zhu L., Tang Z., Qin G., Chen Q., Chen G. (2018). Mechanical properties of gelatin/polyacrylamide/graphene oxide nanocomposite double-network hydrogels. Compos. Sci. Technol..

[72] Schindelin J., Arganda-Carreras I., Frise E., Kaynig V., Longair M., Pietzsch T., Preibisch S., Rueden C., Saalfeld S., Schmid B. (2012). Fiji: An open-source platform for biological-image analysis. Nat. Methods.

[73] Johnston J.S., Bennett M.D., Rayburn A.L., Galbraith D.W., Price H.J. (1999). Reference standards for determination of DNA content of plant nuclei. Am. J. Bot..

[74] Schneider C.A., Rasband W.S., Eliceiri K.W. (2012). NIH Image to ImageJ: 25 years of image analysis. Nat. Methods.

[75] Reyes Ortega F., Rodríguez G., Rosa Aguilar M., García-Sanmartín J., Martínez A., San Román J. (2012). Comportamiento reológico de geles biodegradables para aplicaciones en medicina regenerativa. Soc. Ibér. Biomec. Biomater..

[76] Piao Y., Chen B. (2017). Synthesis and mechanical properties of double cross-linked gelatin-graphene oxide hydrogels. Int. J. Biol. Macromol..

[77] Krishnamoorthy K., Veerapandian M., Yun K., Kim S.J. (2013). The chemical and structural analysis of graphene oxide with different degrees of oxidation. Carbon N. Y..

[78] Sahraei R., Ghaemy M. (2017). Synthesis of modified gum tragacanth/graphene oxide composite hydrogel for heavy metal ions removal and preparation of silver nanocomposite for antibacterial activity. Carbohydr. Polym..

[79] Allah H., Mohamed S.T., Sakhawy E., Kamel S. (2021). Carboxymethyl Cellulose—Grafted Graphene Oxide/Polyethylene Glycol for Efficient Ni (II) Adsorption. J. Polym. Environ..

[80] Das L., Das P., Bhowal A., Bhattachariee C. (2020). Synthesis of hybrid hydrogel nano-polymer composite using Graphene oxide, Chitosan and PVA and its application in waste water treatment. Environ. Technol. Innov..

[81] Sarkar N., Sahoo G., Swain S.K. (2020). Nanoclay sandwiched reduced graphene oxide filled macroporous polyacrylamide-agar hybrid hydrogel as an adsorbent for dye decontamination. Nano-Struct. Nano-Objects.

[82] Zhang X.J., Cai W.B., Hao L.Y., Hu X.H., Wei X.J., Wang X.Y., Lin Q. (2018). Preparation of thermo/pH-sensitive reduced graphene oxide interpenetrating hydrogel nanocomposites for co-delivery of paclitaxel and epirubicin. Mater. Technol..

[83] Kumar A., Zo S.M., Kim J.H., Kim S.C., Han S.S. (2019). Enhanced physical, mechanical, and cytocompatibility behavior of polyelectrolyte complex hydrogels by reinforcing halloysite nanotubes and graphene oxide. Compos. Sci. Technol..

[84] Jafarigol E., Salehi M.B., Mortaheb H.R. (2020). Preparation and assessment of electro-conductive poly(acrylamide-co-acrylic acid) carboxymethyl cellulose/reduced graphene oxide hydrogel with high viscoelasticity. Chem. Eng. Res. Des..

[85] Chang Z., Chen Y., Tang S., Yang J., Chen Y., Chen S., Li P., Yang Z. (2020). Construction of chitosan/polyacrylate/graphene oxide composite physical hydrogel by semi-dissolution/acidification/sol-gel transition method and its simultaneous cationic and anionic dye adsorption properties. Carbohydr. Polym..

[86] Sarkar N., Sahoo G., Swain S.K. (2020). Graphene quantum dot decorated magnetic graphene oxide filled polyvinyl alcohol hybrid hydrogel for removal of dye pollutants. J. Mol. Liq..

[87] Youssef A.M., Hasanin M.S., El-Aziz M.E.A., Turky G.M. (2021). Conducting chitosan/hydroxylethyl cellulose/polyaniline bionanocomposites hydrogel based on graphene oxide doped with Ag-NPs. Int. J. Biol. Macromol..

[88] Jafarigol E., Afshar Ghotli R., Hajipour A., Pahlevani H., Baghban Salehi M. (2021). Tough dual-network GAMAAX hydrogel for the efficient removal of cadmium and nickle ions in wastewater treatment applications. J. Ind. Eng. Chem..

[89] Tarashi S., Nazockdast H., Sodeifian G. (2019). Reinforcing effect of graphene oxide on mechanical properties, self-healing performance and recoverability of double network hydrogel based on κ-carrageenan and polyacrylamide. Polymer.

[90] Shah S.A., Kulhanek D., Sun W., Zhao X., Yu S., Parviz D., Lutkenhaus J.L., Green M.J. (2020). Aramid nanofiber-reinforced three-dimensional graphene hydrogels for supercapacitor electrodes. J. Colloid Interface Sci..

[91] Sharma B. (2020). Viscoelastic investigation of graphene oxide grafted PVA biohybrid using ostwald modeling for packaging applications. Polym. Test..

[92] Hasda A.M., Vuppaladadium S.S.R., Qureshi D., Prasad G., Mohanty B., Banerjee I., Shaikh H., Anis A., Sarkar P., Pal K. (2020). Graphene oxide reinforced nanocomposite oleogels improves corneal permeation of drugs. J. Drug Deliv. Sci. Technol..

[93] Luo H., Dong J., Yao F., Yang Z., Li W., Wang J., Xu X., Hu J., Wan Y. (2018). Layer-by-Layer Assembled Bacterial Cellulose/Graphene Oxide Hydrogels with Extremely Enhanced Mechanical Properties. Nano-Micro Lett..

[94] Nath J., Shekhar S., Dolui S.K. (2021). Artificial Nacre-based Chitosan/Graphene Oxide-Mg Hydrogel with Significant Mechanical Strength and Shape Memory Effect. Polym. Sci. Ser. A.

[95] di Luca M., Vittorio O., Cirillo G., Curcio M., Czuban M., Voli F., Farfalla A., Hampel S., Nicoletta F.P., Iemma F. (2018). Electro-responsive graphene oxide hydrogels for skin bandages: The outcome of gelatin and trypsin immobilization. Int. J. Pharm..

[96] Huang Y., Xiao L., Zhou J., Li X., Liu J., Zeng M. (2020). Mechanical enhancement of graphene oxide-filled chitosan-based composite hydrogels by multiple mechanisms. J. Mater. Sci..

[97] Cheng W., Chen Y., Teng L., Lu B., Ren L., Wang Y. (2018). Antimicrobial colloidal hydrogels assembled by graphene oxide and thermo-sensitive nanogels for cell encapsulation. J. Colloid Interface Sci..

[98] Wang J., Chen G., Zhao Z., Sun L., Zou M., Ren J., Zhao Y. (2018). Responsive graphene oxide hydrogel microcarriers for controllable cell capture and release. Sci. China Mater..

[99] Breuer L., Raue M., Strobel M., Mang T., Schöning M.J., Thoelen R., Wagner T. (2016). Hydrogels with incorporated graphene oxide as light-addressable actuator materials for cell culture environments in lab-on-chip systems. Phys. Status Solidi Appl. Mater. Sci..

[100] Ligorio C., Zhou M., Wychowaniec J.K., Zhu X., Bartlam C., Miller A.F., Vijayaraghavan A., Hoyland J.A., Saiani A. (2019). Graphene oxide containing self-assembling peptide hybrid hydrogels as a potential 3D injectable cell delivery platform for intervertebral disc repair applications. Acta Biomater..

[101] Liu P., Shao H., Kong Y., Wang D. (2020). Effect of graphene oxide exposure on intestinal Wnt signaling in nematode Caenorhabditis elegans. J. Environ. Sci..

