# Supplementary material for: Gelatin-Graphene Oxide Nanocomposite Hydrogels for Kluyveromyces lactis Encapsulation: Potential Applications in Probiotics and Bioreactor Packings
Source: Biomolecules. 2021 Jun 22;11(7):922. doi: 10.3390/biom11070922 (PMC8302002; doi:10.3390/biom11070922)
Supplement: Supplementary file 1 [file biomolecules-11-00922-s001.zip › biomolecules-1241771-supplementary.pdf]

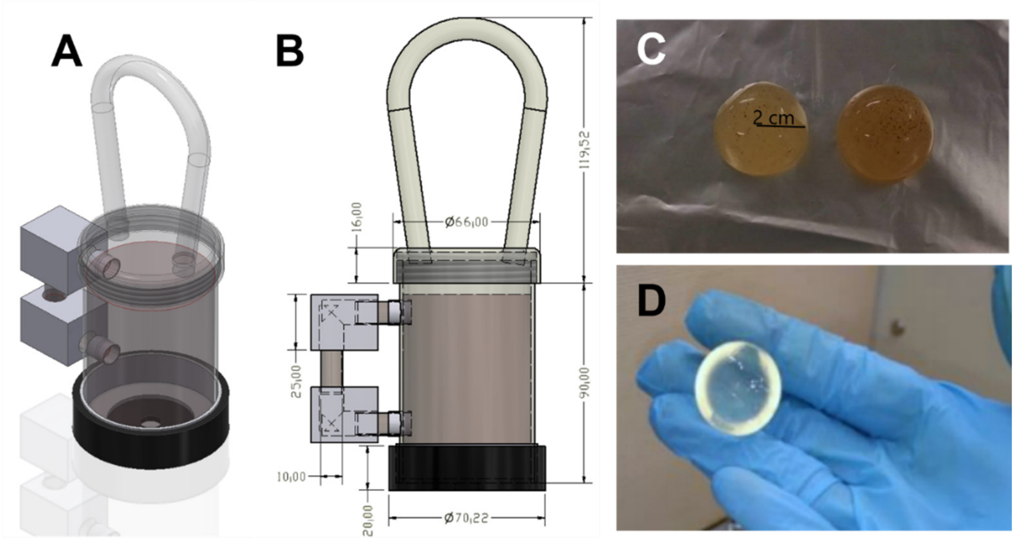

Figure S1. Packed-bed bioreactor designed and 3D-printed for this study. (**A**) 3D model. (**B**) Isometric view (measurements in millimeters). (**C**) Half-sphere graphene oxide (GO) nanocomposite hydrogels used for packing bioreactor. (**D**) Gel sample without GO.
